# Demographic Characteristics, Comorbidities, and Length of Stay of COVID-19 Patients Admitted Into Intensive Care Units in Saudi Arabia: A Nationwide Retrospective Study

**DOI:** 10.3389/fmed.2022.893954

**Published:** 2022-07-13

**Authors:** Maram Al-Otaiby, Khalid M. Almutairi, Jason M. Vinluan, Ahad Al Seraihi, Wadi B. Alonazi, Mohammad Hassan Qahtani, Thamer Aljeri, Manal A. Alhumud, Nadhar Alobaidi, Sultana A. Alhurishi

**Affiliations:** ^1^The Saudi Ministry of Health, Riyadh, Saudi Arabia; ^2^Department of Pathology, College of Medicine, King Saud University, Riyadh, Saudi Arabia; ^3^Department of Community Health Science, College of Applied Medical Sciences, King Saud University, Riyadh, Saudi Arabia; ^4^College of Business Administration, King Saud University, Riyadh, Saudi Arabia

**Keywords:** COVID-19, length of stay, risk factors, ICU admission, Saudi Arabia

## Abstract

**Background:**

This study aimed to describe the demographic characteristics and determine the risk factors associated with disease severity and length of hospital and intensive care unit (ICU) stay in a cohort of COVID-19 patients admitted into ICU in Saudi Arabia.

**Methods:**

This was a national, multi-center, retrospective cross-sectional study of all COVID-19 cases admitted into different ICUs in Saudi Arabia between March 2020 and September 202l. Demographic, clinical features, comorbidities, and length of stay (LOS) data were retrieved from the national Health Electronic Surveillance Network (HESN) and Taqassi databases at the Saudi Ministry of Health (MOH) for subsequent analyses. We used multiple linear regression models to determine risk factors associated with critical outcomes (including LOS in ICU) among COVID-19 cases.

**Results:**

A total of 12,436 COVID-19 patients were included in this study, with a mean age of 59.57 ± 18.30 years and 7,679 (62%) were <65 years old. COVID-19 was more common in males (*N* = 7,686, 61.9%) and Saudi nationals (*N* = 8,516, 68.5%). The clinical characteristic findings showed that 36.3% of patients required invasive ventilation whilst 65.4% received tracheostomies for ventilation, and 4% were on dialysis. Our analysis revealed that 2,978 (23.9%) patients had one comorbidity, 4,977 (47.4%) had two or more comorbidities, and diabetes (48.2%) was the most prevalent comorbidity, followed by hypertension (44.2%), and chronic cardiovascular disease (10.5%). Thirteen variables emerged as significant predictors of LOS in ICU using multiple linear regression analyses, with invasive ventilation as the strongest predictor of LOS in the ICU (beta = −0.68, *p* = 0.001) and hospital admission (beta = −0.65, *p* = 0.001).

**Conclusions:**

COVID-19 continues to affect millions of people around the world, with a mortality rate of about 2–3% of all infected patients. Our analysis revealed that comorbidities such as chronic kidney disease, cardiovascular disease, diabetes, and older age were significant risk factors associated with a poorer prognosis and longer duration of stay in hospitals and ICU.

## Introduction

The coronavirus disease (COVID-19) pandemic has caused a serious threat and catastrophes worldwide. The first case of COVID-19 in Saudi Arabia was identified on the 2nd of March 2020 by the Ministry of Health (MOH) and, as of January 31st, 2022, there had been 687,264 confirmed cases with COVID-19 and 8,940 deaths (1). Different hospitals within the Kingdom were at maximum capacity due to the high number of patients admitted with COVID-19 and their notable length of stay (LOS). Indeed, Saudi Arabia is considered the most affected country in the Gulf Cooperation Council (GCC) region (2).

Several studies have indicated that the clinical severity of and mortality from COVID-19 are heterogeneous and vary depending on both demographic and clinical characteristics (3). For example, Richardson et al. (4) reported that hypertension, obesity, and diabetes were the most common comorbidities among hospitalized COVID-19 patients in the US. Similarly, in China, older male patients with pre-existing cardiovascular disease, diabetes, asthma, and chronic lung disease had higher mortality rates (5, 6). In Italy and the United Kingdom, however, cardiovascular disease, hypertension, and obesity were associated with a more adverse course of COVID-19 (7, 8).

An additional impact of the COVID-19 pandemic was the increased demand for hospital beds and shortages in healthcare staff and equipment. Indeed, the influx of patients needing hospitalization raised serious concerns regarding the burden of the pandemic on healthcare providers and the healthcare system. Therefore, identifying risk factors associated with increased hospital care demand, such as the type of patients requiring hospitalization and the duration of hospitalization, can be useful for preparedness and decision making (9, 10). However, the LOS of COVID-19 patients differs depending on symptom severity and can also vary between general wards and intensive care units (ICU), where patients may need advanced critical care such as additional oxygen support or mechanical ventilation (9, 11).

There are limited studies on the clinical manifestations and LOS of hospitalized ICU patients with COVID-19 in Saudi Arabia. Knowledge of these factors would help to triage high-risk patients and prioritize ICU admissions. Furthermore, it would help in pandemic control, and minimize the burden on the healthcare system. Therefore, here we assessed the risk factors associated with ICU admission among COVID-19 patients in Saudi Arabia, incorporating demographic characteristics, LOS, comorbidities, and clinical outcomes. In particular, we estimated whether COVID patients' LOS differed between those who were admitted into ICU and hospital admission.

## Methods

### Study Design and Setting

This retrospective cross-sectional multi-center cohort study examined the demographic and clinical characteristics of all COVID-19 positive cases admitted into ministry of health (MOH) hospitals across the Kingdom of Saudi Arabia between March 1, 2020, and September 15, 2021. This study has been ethically approved by the Institutional Review Board (IRB) committee from the General Department of Research and Studies (GDRS) at MOH (IRB Log No. 21-108M). Data privacy and confidentiality were maintained throughout the study. Informed consent was waived due to the retrospective and observational nature of the study.

### Data Collection

Data were mainly derived from two databases. The first was the Health Electronic Surveillance Network (HESN), an electronic surveillance system launched by the Saudi MOH that provides accurate public health information to healthcare professionals and decision-makers (12). The second system was Taqassi, which is concerned with the investigation of infectious and epidemic diseases. The HESN and Taqassi databases were both utilized as secondary data sources and cover the entire healthcare facilities within Saudi Arabia. Reporting of confirmed COVID-19 cases by these facilities has been mandatory since the start of the pandemic. The routinely collected data from HESN and Taqassi databases included patient demographics (e.g., age, gender, and nationality), hospital diagnoses, ICU status, treatments (including invasive mechanical ventilation and kidney replacement therapy), comorbidity status (having no comorbidity or suffering from one or more of the following: asthma, chronic kidney disease, chronic lung disease, chronic cardiovascular disease, diabetes, hypertension, hemoglobin disorder, or liver disease), and outcomes (including LOS, discharge, re-admission, and/or mortality). The primary outcome of our study was the ICU and hospital LOS in days, whilst the secondary outcomes were the survival status (ICU or hospital admission, recovery, or death).

### Study Participants

All adult patients aged 18 years old and/or older who were admitted into the ICU at MOH hospitals with COVID-19 between March 1, 2020 and September 15, 2021, were included in this study. A diagnosis of COVID-19 was confirmed based on a positive test result of quantitative reverse transcription polymerase chain reaction (RT-PCR) performed on nasopharyngeal swabs. Additionally, to confirm COVID-19 diagnosis, only COVID-19 test swabs taken within 14 days prior to and 1 day post-hospital admission were included in the study. Moreover, to obtain a precise estimate of the LOS, hospital admissions for health reasons other than COVID 19 were excluded from the study. In addition, patients under 18 years old, those with multiple ICU entries, or records with missing data and/or data of unidentified IDs were excluded from the study. Study participants were further sub-categorized into survivors (*n* = 8,210) and non-survivors (*n* = 4,226). Survivors were defined as recovered ICU patients discharged from hospital or transferred to another ward, whereas non-survivors were defined as patients who died during the study period.

### Statistical Analysis

Data were represented using descriptive statistics including frequency and percentage tables, means, and standard deviations (SD). The normality of the data was evaluated using the Kolmogorov–Smirnov test. Moreover, student's *t*-test and analysis of variance (ANOVA) were used for normally distributed variables between groups, whilst Mann-Whitney U and Kruskal–Wallis tests were conducted for non-normally distributed variables accordingly. The Chi-square test were applied to determine statistically significant differences between ICU COVID-19 status and other categorical variables. The LOS in the ICU and hospital admission were logarithmically transformed prior to all parametric analyses. Multiple linear regression models were performed to examine the impact of age, gender, and different comorbidities on the recovery period, LOS in ICU and hospital admission. In addition, the Charlson Comorbidity Index (CCI) was assessed to predict a 10-year survival rate in patients with comorbidities and was used as a measure of the total comorbidity burden (13). The SPSS v.23 (IBM Statistics, Armonk, NJ) tool was used for statistical data analysis. Statistical significance was determined at a *p*-value of <0.05.

## Results

### Baseline Characteristics of the Study Population

A total of 12,436 patients were included in the study, admitted between March 2020 and September 2021. Table 1 represents the baseline demographic and clinical characteristics of the study cohort. The mean age was 59.57 ± 18.30 years, and 2267 patients (18.2%) were more than 75 years old. The gender distribution of our patients was 4,750 (38.2%) females and 7,686 (61.8%) males, and 8,516 (68.5%) were Saudi nationals. The median ICU LOS was 6 days [interquartile range (IQR) 0–398 days] and the average ICU LOS was 9.4 ± 14.3 days, while the median hospital admission LOS was 10 days (IQR 0–403 days) and the average hospital LOS was 13.8 ± 15.9 days. Only 36.3% (*n* = 4,509) of patients received invasive ventilation, while 65.4% (*n* = 8,131) were given a tracheostomy for ventilation. Interestingly, only few patients (*n* = 499; 4%) required renal replacement treatment (dialysis) and 17.4% (*n* = 2,167) had more than one ICD-10 diagnosis. Precisely, out of the 12,436 COVID-19 ICU patients, 2,978 (23.9%) had one comorbidity while 3,062 (24.6%) had three or more comorbidities simultaneously. Of these 12,436 patients, 4,226 died (34%) at the study endpoint (denoted by non-survivors), whilst 8,210 (66%) recovered or remained in the ICU (denoted by survivors).

**Table 1:** Demographic and clinical characteristics of COVID-19 cases in ICU.

**Characteristic**	**Total *n* = 12,436**	**Survivors *n* = 8210 (66%)**	**Non-survivors *n* = 4,226 (34%)**	***p*-value**
**Age**				**0.001**
Mean—SD	59.57 ± 18.30			
19–44	2,316 (18.6)	1,877 (22.9)	439 (10.4)	
45–54	2,334 (18.8)	1,736 (21.1)	598 (14.2)	
55–64	3,029 (24.4)	1,993 (24.3)	1,036 (24.5)	
65–74	2,490 (20.0)	1,472 (17.9)	1,018 (24.1)	
More than 75 years old	2,267 (18.2)	1,132 (13.8)	1,135 (26.9)	
**Gender**				0.246
Male	7,686 (61.8)	5,056 (65.8)	2,630 (34.2)	
Female	4,750 (38.2)	3,154 (66.4)	1,596 (33.6)	
**Nationality**				**0.038**
Saudi	8,516 (68.5)	5,578 (65.5)	2,938 (34.5)	
Non-Saudi	3,920 (31.5)	2,632 (67.1)	1,288 (32.9)	
**No of days in the ICU** Median (minimum—maximum)	6 (0–398)	3 (0–390)	11 (0–398)	**0.001**
**No of days in the ICU** Mean—SD	9.41 ± 14.33	14.34 ± 15.53	6.86 ± 12.96	
**No of days in hospital admission** Median (minimum—maximum)	10 (0–403)	8 (0–390)	14 (0–403)	**0.001**
**No of days in hospital admission** Mean—SD	13.79 ± 15.90	11.54 ± 14.92	16.84 ±15.90	
**Invasive ventilation**				**0.001**
Yes	4,509 (36.3)	1,580 (35.0)	2,929 (65.0)	
No	7,927 (63.7)	6,630 (83.6)	1,297 (16.4)	
**Type of invasive ventilation**				**0.001**
Tracheostomy	8,131 (65.4)	6,750 (83.0)	1,381 (17.0)	
Endotracheal tube	4,305 (34.6)	1,460 (33.9)	2,845 (66.1)	
**Type of non-invasive ventilation**				**0.001**
BIPAP	737 (5.9)	484 (65.7)	253 (34.4)	
CIPAP	337 (2.7)	191 (57.0)	144 (43.0)	
High flow canula	1,317 (10.6)	1,065 (80.9)	252 (19.1)	
Venturi mask	56 (0.5)	43 (76.8)	13 (23.2)	
None	9,991 (80.3)	6,427 (64.3)	3,564 (35.7)	
**Oxygen therapy**				**0.001**
Face mask	1,789 (14.4)	1,613 (90.2)	176 (9.8)	
Nasal canula	1,308 (10.5)	1,222 (93.4)	86 (6.6)	
Non-rebreathing mask	1,613 (13.0)	1,318 (81.7)	295 (18.3)	
Venturi mask	25 (0.2)	15 (60.0)	10 (40.0)	
None	7,701 (61.9)	4,042 (52.5)	3,659 (47.5)	
**ECMO**				0.216
Yes	82 (0.7)	58 (70.7)	24 (29.3)	
No	12,354 (99.3)	8,152 (68.0)	4,202 (34.0)	
**Plasmapheresis**
Yes	0			—
No	12,436 (100)	8,210 (66.0)	4,226 (34.0)	
**Renal replacement (dialysis)**				**0.001**
Yes	499 (4.0)	203 (40.7)	296 (59.3)	
No	11,946 (96.0)	8,007 (67.1)	3,939 (32.9)	
**Diagnosis ICD-10 short description**				0.307
1	10,269 (82.6)	6,790 (66.1)	3,479 (33.9)	
More than 1	2,167 (17.4)	1,420 (65.5)	747 (34.5)	
**Comorbidities**				**0.001**
None	3,566 (28.7)	2,686 (75.3)	880 (24.7)	
1	2,973 (23.9)	2,022 (68.0)	951 (32.0)	
2	2,835 (22.9)	1,795 (63.3)	1,040 (36.7)	
3 or more	3,062 (24.6)	1,707 (55.7)	1,355 (44.3)	
**Charlson comorbidity index (CCI)** Median	1 (0–2)	1 (0–2)	2 (1–4)	**0.001**
0 point	4,301 (34.6)	3,127 (38.1)	1,174 (27.8)	
1 point	3,131 (25.2)	2,118 (25.8)	1,013 (24.0)	
2 points	3,376 (27.1)	2,088 (25.4)	1,288 (30.5)	
More than 2 points	1,628 (13.1)	877 (10.7)	751 (17.8)	

*No. of days in ICU, t = −28.39; No. of days in hospital, t = 16.98; R^2^ = 0.997; Age (N = 41, 0.3% missing); p-values in bold are significant at <0.05*.

### Differences Between Survivors and Non-survivors

Remarkably, there were significant differences in the demographic and clinical characteristics between survivors and non-survivors (Table 1). For example, patients who were younger were more likely to survive compared to older patients (aged 19–44, 22.9% vs. more than 75 years old, 13.8%), contrasting with non-survivors who were mostly elderly (aged 19–44, 10.4% vs. more than 75 years old, 26.9%) (*p*-value = 0.001). Notably, whilst only 19.2% (*n* = 1,580) of survivors received invasive mechanical ventilation, the majority of non-survivors (*n* = 2,929; 69.3%) received it, whereby 2,845 (67.3%) were administered endotracheal tube ventilation and 1,381 (32.7%) received tracheostomy (*p*-value < 0.001). Moreover, non-survivors were more frequently treated with renal replacement therapy (dialysis) (*n* = 296; 59.3%) than survivors (*n* = 203; 40.7%; *p*-value < 0.001). It is worth mentioning, however, that non-survivors experienced a significantly longer LOS compared with survivors, with a median LOS in ICU of 11 vs. 3 days (*p*-value = 0.001) and median total hospital LOS of 14 vs. 8 days (*p*-value = 0.001), respectively. Furthermore, 1,355 (32%) of non-survivors experienced three or more comorbidities compared with only 1,707 (20.8%) of survivors. Interestingly, the median CCI score was higher in the non-survivor (2, IQR 1–4) compared with the survivor group (1, IQR 0–2). In particular, 17.8% of non-survivors had a CCI score of more than 2 points, contrasting with 10.7% of the survivors (Table 1), potentially reflecting the severity of disease and associated comorbidities in the non-survivor group.

### Associations Between Demographic Variables and LOS

**Figure 1:**
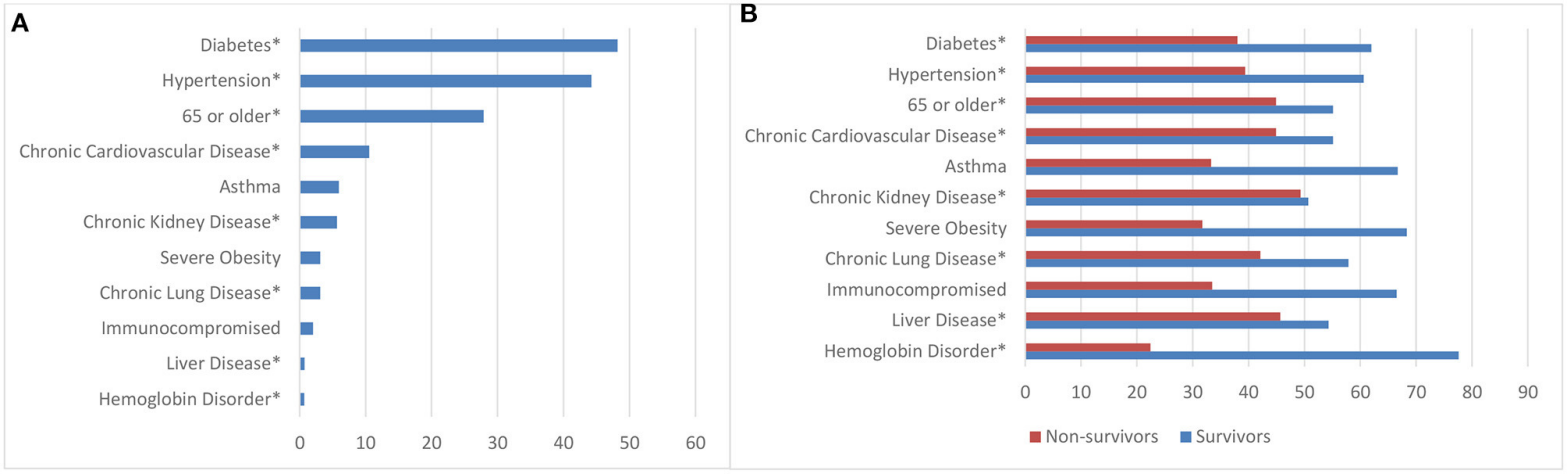
Frequency distribution of comorbidities of COVID-19 ICU patients and comparison between survivors and non-survivors. **(A)** Overall frequency distribution of comorbidities of COVID-19 ICU patients. **(B)** Comparison of comorbidities between survivors and non-survivors. *p-value significant at <0.05.

**Figure 2:**
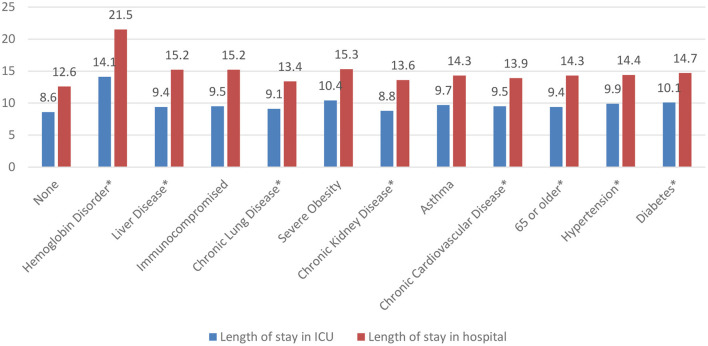
Length of ICU and hospital stay of COVID-19 patients in days by comorbidity. *p-value significant at <0.05.

To determine the frequency of comorbidities observed in our COVID-19 ICU patients, diabetes was the most common comorbidity at presentation (48.2%; *n* = 5,995), followed by hypertension (44.2%; *n* = 5,501), aged 65 or older (27.0%, *n* = 3,469), and chronic cardiovascular disease (10.5%; *n* = 1,311) (Figure 1A). Remarkably, Figure 1B revealed that survivors and non-survivors differed significantly in terms of their comorbidities. However, to determine which comorbidities were associated with a significantly longer length of ICU and hospital stay, patients with hemoglobin disorders had the highest average LOS in ICU (14.1 ± 19.2 days), followed by those with severe obesity (10.4 ± 14.7 days) and diabetes (10.1 ± 15.4 days). Similarly, patients with hemoglobin disorder also had the highest average hospital LOS (21.5 ± 19.9 days) followed by severe obesity (15.3 ± 15.8 days) and liver disease (15.2 ± 13.4 days) (Figure 2).

One-way ANOVA analysis was then conducted to further explore associations between the number of comorbidities and LOS in ICU and hospital admission. Table 2 demonstrates that COVID-19 patients with two or more comorbidities had significantly longer LOS in ICU (10.0 ± 15.4 days) and hospital admission (14.4 ± 16.8 days) compared with patients with only one comorbidity (*p*-value = 0.001).

**Table 2:** One-way ANOVA analysis of COVID-19 patients' length of stay in ICU and hospital admission in days by number of comorbidities.

**Number of comorbidities**	**Length of stay in ICU**		**Length of stay in hospital**	
	**Cases with comorbidity (days, mean ±SD)**	***p*-value (one way ANOVA)**	**Cases with comorbidity (days, mean ±SD)**	***p*-value (one way ANOVA)**
None	8.6 ± 14.5	**0.001**	12.6 ± 15.9	**0.001**
1	9.5 ± 12.4		13.8 ± 13.4	
2	10.0 ± 15.4		14.4 ± 16.8	
3 or more	9.7 ± 14.8		13.8 ±15.6	

*Length of hospital stay in ICU df = 3, F = 10.86; Hospital admission df = 3, F = 18.04. p-values in bold are significant at <0.05*.

Finally, we investigated the risk factors and predictors of LOS in ICU and hospital admission, adjusting for demographics, clinical characteristics, and comorbidities using a multiple linear regression model. Preliminary analyses were conducted to ensure no violations of normality, linearity, and multicollinearity (Supplementary Figure 1). The total variance explained by the model in LOS in ICU and demographic characteristics was 12.3%, *F*_(20, 9, 621)_ = 67.24, *p*-value < 0.001. In the final model of LOS in ICU, thirteen variables emerged as significant predictors of LOS in ICU as shown in Table 3. These were age, invasive ventilation, the type of ventilation (tracheostomy and/or endotracheal tube), renal replacement therapy, diagnosis of ICD-10, ICU status (survivors and non-survivors), chronic kidney disease, chronic lung disease, cardiovascular disease, diabetes, hemoglobin disorder, number of comorbidities and CCI (*p*-value < 0.05). In contrast, the total variance explained by the model of LOS in hospital admission was 13.3%, *F*_(20, 9, 728)_ = 74.41, *p*-value 0.001. Compared with LOS in ICU, twelve predictors were associated with LOS in hospital admission (Table 3). These include age, invasive ventilation, the type of ventilation (tracheostomy and endotracheal tube), renal replacement therapy, diagnosis of ICD-10, ICU status (survivors and non-survivors), asthma, diabetes, hypertension, hemoglobin disorder, number of comorbidities and CCI (*p*-value < 0.05). Of note, invasive ventilation constituted the strongest predictor of LOS in ICU (beta = −0.68, 95% CI −0.66 to 0.53, *p*-value = 0.001), and hospital admission (beta = −0.65, 95% CI −0.60 to 0.48, *p*-value = 0.001) (Table 3).

**Table 3:** Multiple linear regression analyses for the length of stay in ICU and hospital admissions adjusted for demographic variables, clinical characteristics, and comorbidities.

**Variable**	**Length of stay in ICU**		**Length of stay in hospital**	
	***B* (95% CI)**	***p*-value**	***B* (95% CI)**	***p*-value**
Age	0.03 (0.01 to 0.03)	**0.001**	0.05 (0.01 to 0.04)	**0.001**
Gender	0.01 (−0.01 to 0.20)	0.829	0.01 (−0.01 to 0.20)	0.568
Nationality	0.24 (0.02 to 1.09)	0.062	0.01 (−0.01 to 0.03)	0.051
Invasive ventilation	−0.68 (−0.66 to −0.53)	**0.001**	−0.65 (−0.60 to −0.48)	**0.001**
Type of ventilation	−0.55 (−0.56 to −0.31)	**0.001**	−0.53 (−0.51 to −0.39)	**0.001**
Renal replacement	−0.03 (−0.11 to −0.02)	**0.002**	−0.04 (−0.12 to −0.04)	**0.001**
Diagnosis ICD-10 Short description	−0.04 (−0.06 to −0.02)	**0.001**	−0.03 (−0.05 to 0.02)	**0.001**
ICU status	0.17 (0.12 to 0.16)	**0.001**	−0.18 (0.13 to 0.178)	**0.001**
Asthma	−0.01 (−0.04 to 0.03)	0.724	0.04 (0.04 to 0.11)	**0.001**
chronic kidney disease	−0.13 (−0.30 to −0.18)	**0.001**	−0.02 (−0.08 to 0.01)	0.050
Chronic lung disease	−0.02 (−0.12 to 0.02)	**0.006**	−0.01 (−0.04 to 0.05)	0.927
Chronic cardiovascular disease	−0.08 (−0.14 to 0.08)	**0.001**	−0.01 (−0.04 to 0.02)	0.553
Diabetes	0.06 (0.03 to 0.07)	**0.001**	0.10 (0.05 to 0.11)	**0.001**
Hypertension	−0.01 (−0.457 to 0.01)	0.288	0.07 (0.03 to 0.08)	**0.001**
Immunocompromised	0.02 (−0.01 to 0.12)	0.044	0.02 (0.02 to 0.15)	0.005
Hemoglobin disorder	0.05 (0.18 to 0.39)	**0.001**	0.04 (0.14 to 0.34)	**0.001**
Liver disease	−0.01 (−0.19 to 0.01)	0.033	0.01 (0.01 to 0.18)	0.061
No of comorbidities	−0.17 (−0.08 to −0.03)	**0.001**	−0.05 (−0.06 to −0.02)	**0.001**
CCI	0.03 (0.01 to 0.05)	**0.001**	0.05 (0.01 to 0.03)	**0.001**

*p-values in bold are significant at <0.05*.

## Discussion

This national, multi-center, retrospective study provides an in-depth description of the demographic and clinical characteristics of ICU patients with COVID-19 in Saudi Arabia and highlights the risk factors associated with increased LOS in ICU and hospital admission. Identifying these predictors may help provide appropriate medical interventions and improvements in clinical care to enhance outcomes for our COVID-19 patients.

In this large sample of 12,436 critically ill patients with COVID-19, we reported a high mortality rate of ~34% at the study endpoint. This outcome is consistent with recent studies conducted in the United Kingdom and Italy, which found that 38–46% of 3,988 and 12,133 patients admitted into the ICU, respectively, died from COVID-19 (8, 14). The mean age of our patients was 59.6 years, similar to studies carried out in the United States and Italy (4, 8). We also found that males were more affected by COVID-19 than females and were more likely to need mechanical ventilation, akin to findings from previous studies in other countries (15–19). Interestingly, ~22.9% (*n* = 2,835) of our cohort had two or more comorbidities, mirroring Saudi studies by Alsofayan et al. (3) and Khan et al. (20) which reported a similar rate (20.1–29%). Most importantly, our findings revealed that diabetes was the most prevalent comorbidity (48.2%) followed by hypertension (44.2%) and older age (27.9%). Indeed, older age, diabetes, and kidney disease have a negative impact on innate immunity and are considered risk factors associated with mortality from COVID-19, warranting extra vigilance and care of this vulnerable patient population (21).

In our study, non-survivors experienced a significantly longer LOS in ICU compared with survivors (11 vs. 3 days), consistent with previous findings from China showing that the average LOS in hospital was 11 and 21 days in ICU (22). In a US study, LOS was 5 days for non-severe cases and 15 days for severe cases requiring ICU admission (23). An important observation from our study was that having two or more comorbidities increased the risk of hospitalization and LOS in ICU and hospital admission. In particular, patients with hemoglobin disorder and severe obesity had a significantly longer LOS in ICU and hospital admission compared with other comorbidities or having a single comorbidity. These findings highlight the impact of comorbidities on COVID-19 severity, in line with previously published studies (21, 24–26), and indicate that the number of comorbidities may influence prognosis and COVID-19-related LOS in ICU and hospital admissions. Moreover, the association between certain comorbidities and poor outcomes may be partly due to their effect on immunity. For example, in hemoglobin disorders, anemia may cause pulmonary capillary leak syndrome, resulting in acute respiratory distress syndrome (ARDS) (27, 28). Indeed, a hematological disorder together with other comorbidities such as cardiovascular and respiratory diseases should be taken into consideration with extra care because of the higher mortality associated with these conditions (29). Such action may lessen potential complications that may arise to COVID-19 ICU patients.

This study, however, has some limitations. First, due to the retrospective nature of the data collection, only variables that were routinely collected were included in the study, including clinical data retrieved from the patients' medical records, and so certain laboratory and radiological findings were not investigated. Second, the impact of different treatment modalities on clinical outcomes and LOS was not studied. Nevertheless, this retrospective national study provides new insights into the clinical characteristics and outcomes of hospitalized patients with COVID-19 across the Kingdom, which will be useful for mapping the illness, risk-stratifying patients, and directing effective public health measures and policies. Further research is needed to clarify if the LOS differs between COVID-19 vaccinated and unvaccinated ICU cases. We therefore recommend further investigation into the relative survival of COVID-19 and its variations by vaccination status.

In conclusion, this large study of hospitalized patients with COVID-19 in Saudi Arabia examined risk factors that could contribute to longer LOS in ICU, severe outcomes, and death. Our findings revealed that having two or more comorbidities (diabetes, hypertension, older age (65 years and above), hemoglobin disorder, chronic kidney disease, and cardiovascular diseases) contributed to a poorer prognosis and a longer length of stay in ICU and hospitals. We believe our results will help decision-makers implement regulations to prognosticate COVID-19 patients, thereby mitigating against the burden of COVID-19 on the healthcare system, and prioritizing the management of patients with comorbidities to protect this vulnerable patient population.

## Data Availability Statement

The data that support the findings of this study are available at the Saudi Ministry of Health (MOH), but restrictions apply to the availability of these data, which were used under license for the current study, and so are not publicly available. Data are however available from the authors upon reasonable request and with permission from the Saudi MOH.

## Ethics Statement

This study involving human participants was reviewed and approved by the Institutional Review Board (IRB) of the General Department of Research and Studies (GDRS) at MOH (IRB Log No. 21-108M). The Ethics Committee waived the requirement of written informed consent for participation.

## Author Contributions

MA-O, KMA, and WBA: supervision. MA-O, KMA, WBA, AA, JMV, MHQ, TA, MAA, NA, and SAA: conceptualization and writing—review and editing. TA, AA, MHQ, NA, and JMV: data curation. KMA and JMV: data analysis. AA and JMV: writing—original draft. MA-O and KMA: funding acquisition. All authors reviewed the results and approved the final version of the manuscript.

## Funding

This study was supported by the Saudi Ministry of Health (MOH) and Researchers Supporting Project (RSP-2022R463) at King Saud University in Riyadh, Saudi Arabia.

## Conflict of Interest

The authors declare that the research was conducted in the absence of any commercial or financial relationships that could be construed as a potential conflict of interest.

## Publisher's Note

All claims expressed in this article are solely those of the authors and do not necessarily represent those of their affiliated organizations, or those of the publisher, the editors and the reviewers. Any product that may be evaluated in this article, or claim that may be made by its manufacturer, is not guaranteed or endorsed by the publisher.
