# Supplementary material for: Demographic Characteristics, Comorbidities, and Length of Stay of COVID-19 Patients Admitted Into Intensive Care Units in Saudi Arabia: A Nationwide Retrospective Study
Source: Front Med (Lausanne). 2022 Jul 13;9:893954. doi: 10.3389/fmed.2022.893954 (PMC9325959; doi:10.3389/fmed.2022.893954)

Supplementary Material

Supplementary Figure 1. The Bland Altman plot (after log10 transformation).

A scatter plot showing the differences between LOS in the ICU and hospital admission plotted against the mean of these two measurements. Values which lie within ±1.96 SD (green lines) of the mean (red line) are considered to be in agreement. The mean differences in LOS prediction for all patients was (0.09, SD 0.16); the prediction upper limit = 0.42 and lower limit = -0.23.

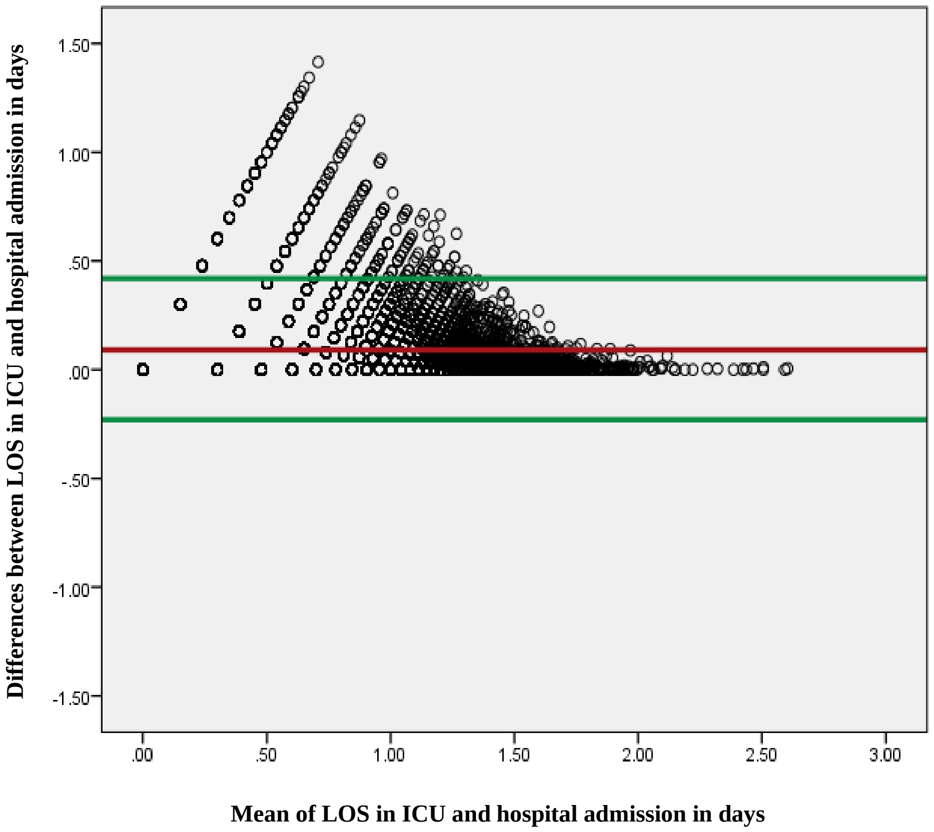

Supplement: Supplementary file 1 [file Data_Sheet_1.pdf]
